# Deposition of Chitosan on Plasma-Treated Polymers—A Review

**DOI:** 10.3390/polym15051109

**Published:** 2023-02-23

**Authors:** Alenka Vesel

**Affiliations:** Department of Surface Engineering, Jozef Stefan Institute, Jamova cesta 39, 1000 Ljubljana, Slovenia; alenka.vesel@guest.arnes.si

**Keywords:** polymer surfaces, chitosan, coatings, plasma-surface modification, adhesion

## Abstract

Materials for biomedical applications often need to be coated to enhance their performance, such as their biocompatibility, antibacterial, antioxidant, and anti-inflammatory properties, or to assist the regeneration process and influence cell adhesion. Among naturally available substances, chitosan meets the above criteria. Most synthetic polymer materials do not enable the immobilization of the chitosan film. Therefore, their surface should be altered to ensure the interaction between the surface functional groups and the amino or hydroxyl groups in the chitosan chain. Plasma treatment can provide an effective solution to this problem. This work aims to review plasma methods for surface modification of polymers for improved chitosan immobilization. The obtained surface finish is explained in view of the different mechanisms involved in treating polymers with reactive plasma species. The reviewed literature showed that researchers usually use two different approaches: direct immobilization of chitosan on the plasma-treated surface or indirect immobilization by additional chemistry and coupling agents, which are also reviewed. Although plasma treatment leads to remarkably improved surface wettability, this was not the case for chitosan-coated samples, where a wide range of wettability was reported ranging from almost superhydrophilic to hydrophobic, which may have a negative effect on the formation of chitosan-based hydrogels.

## 1. Introduction

**Figure 1:**
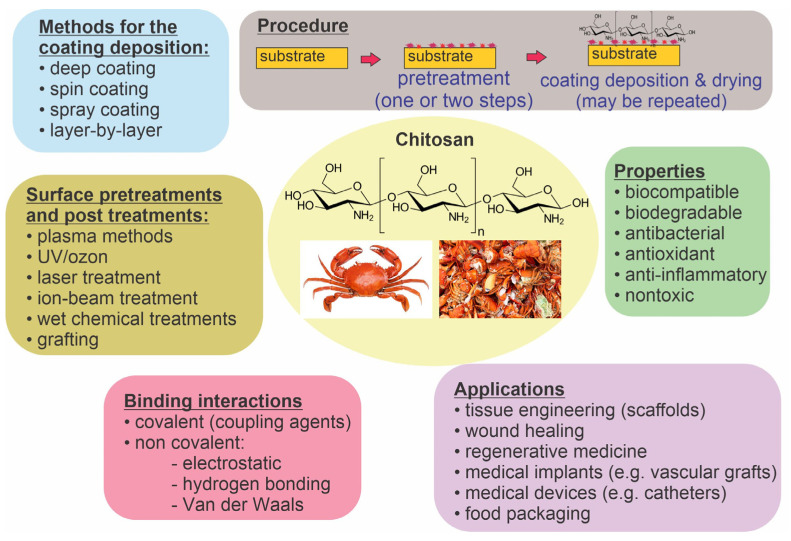
Chitosan’s characteristics, possible applications, and methods of coating preparation.

The surface properties of many polymer materials are inadequate for application in biology or medicine; therefore, they need to be altered [1,2,3,4]. Examples of medical applications are polymeric medical devices such as catheters, which are often the main reason for hospital infections of patients because they provide a suitable habitat for the growth of microbial biofilms [5,6,7,8]. Other examples of medical applications of polymers are polymeric implants such as vascular grafts and bone scaffolds, which should be biocompatible and antithrombogenic to prevent inflammation, restenosis, and thrombosis [9,10]. Polymers can also be used for biomedical packaging when they have to meet special criteria regarding gas and water permeability and ensure long-term sterility [11]. The deposition of coatings with improved biocompatibility, hemocompatibility, and antibacterial properties is particularly interesting [4,12,13,14]. Various coatings have been probed, including natural polysaccharides [15,16,17]. Among them, chitosan is predominantly useful. It can be extracted by treating the chitin shells of crustaceans with an alkaline substance and is abundant in nature [18,19,20,21]. Chitosan has pronounced biocompatibility and biodegradability, as well as antibacterial, antioxidant, anti-inflammatory, and nontoxicity properties [22,23,24,25,26,27]. Furthermore, chitosan-based biomaterials have great potential in tissue engineering and regenerative medicine (i.e., chitosan-based scaffolds), drug delivery and gene therapy (chitosan microspheres), wound healing, and cosmetics [23,28,29,30]. More information about chitosan applications can be found in several recent review papers [23,28,31,32,33]. Its structure, chemical properties, and solubility were also reviewed in [34]. Chitosan can be coupled with other active substances to form various chitosan conjugates, which are reviewed in [35]. It was also reported that chitosan conjugates (with, e.g., polyphenols) exhibit higher antimicrobial activity than films containing only chitosan [36]. Chitosan properties can also be improved through its synthetic modification to form various chitosan derivates with specific functional groups [37]. The most common chitosan derivative is quaternary ammonium chitosan. Different mechanisms of chitosan antibacterial action have been proposed, but the exact mechanism is still not known [38,39]. The future perspective of chitosan can also be intelligent chitosan hydrogels [40]. Figure 1 summarizes chitosan’s characteristics, possible applications, and methods of preparation, which are discussed in more detail further along in this paper.

Polymer materials are hydrophobic, which prevents good adhesion and uniform spreading of the deposited coating over the polymer surface. Therefore, polymer surfaces must first be activated with specific functional groups to ensure good adhesion of chitosan coatings. Chitosan consists of a polysaccharide network with amine NH_2_ groups, which can be protonated in an acidic environment, forming NH_3_^+^ [41]. The positively charged amine groups are suitable for electrostatic binding to negatively charged molecules [42]. Many synthetic polymers that are used for application in biomedicine exhibit a lack of negatively charged surface functional groups such as carboxyl groups. Therefore, chitosan will not stick well to the surface of such polymers. Adhesion of chitosan on polymers can be thus achieved by grafting the substrates with negatively charged molecules [43,44]. Various techniques are available for surface modification and establishing a dense and reasonably uniform distribution of surface functional groups, as well as modification of surface roughness, which may also have a beneficial effect on adhesion because of mechanical interlocking [2,45,46]. These methods include dry techniques such as plasma treatment, laser treatment, ion implantation, ultraviolet/ozone (UV/O_3_) treatment [2,45], and wet chemical treatment using oxidizing wet chemicals [46,47], which has an ecological drawback. Laser treatment causes rippling and formation of periodic surface structures. Laser radiation can break chemical bonds, which leads to the formation of reactive radicals that react with the surrounding atmosphere and form new functional groups [2]. Similarly, UV/ozone treatment also leads to bond dissociation through the absorption of UV photons, which react with ozone [45]. UV treatment followed by UV irradiation can also be used in combination with the exposure of a sample to a photo-initiator [46]. At the same time, ion implantation leads to the breaking of polymer chains and consequently to increased concentration of highly reactive radicals and changes in surface morphology [2]. 

The present review focuses on plasma methods for polymer functionalization for better chitosan adhesion. The paper is divided into four sections. The first describes the role of plasma reactive species and possible surface reaction mechanisms. The second shows a tabular summary of publications on different plasma methods for the immobilization of chitosan and the main results. All publications summarized in this section are then discussed in more detail in Section 4 and Section 5, dealing with direct or indirect immobilization of chitosan on plasma-treated substrates, respectively.

## 2. Surface Functionalization Using Plasma Methods

Nonthermal plasma modification of materials has become a popular technique for surface functionalization [48,49]. Nonequilibrium gaseous plasma consists of free electrons and positively charged ions. The concentration of electrons and positive ions in nonequilibrium plasma is usually equal, except in the plasmas of electronegative gases, where the concentration of positively charged ions is larger than that of free electrons. In any case, the density of free electrons in gaseous plasma is high, usually above 10^14^ m^−3^ [50,51,52]. The free electrons have much higher velocities than any other gaseous plasma species (neutral molecules, radicals, and positively or negatively charged molecules). Therefore, they reach the surface of any object immersed in the nonequilibrium plasma immediately after igniting the discharge, before the other gaseous species. Consequently, substrates exposed to plasma will thus acquire a negative surface charge after even a very short plasma treatment [53,54]. Free electrons, which have accumulated on the polymer surface after igniting the discharge, will not contribute to providing the binding sites for electrostatic interactions with chitosan. Instead, other reactive plasma species should obtain negatively charged functional groups on the polymer surface to benefit from electrostatic interaction with chitosan. 

Negatively charged surface functional groups will appear on a polymer surface upon the chemical interaction of gaseous plasma species with the polymer surface. The most desirable groups are those containing oxygen, such as COOH groups. They will interact with positively charged molecules or a part of a molecule with positively charged regions (such as amino groups in the chitosan). Often, covalent bonds may be formed between the functional groups generated on the polymer surface and the deposited chitosan [42]. The surface functional groups for the immobilization of chitosan may be formed in plasma through one or more mechanisms, as mentioned below.

**Figure 2:**
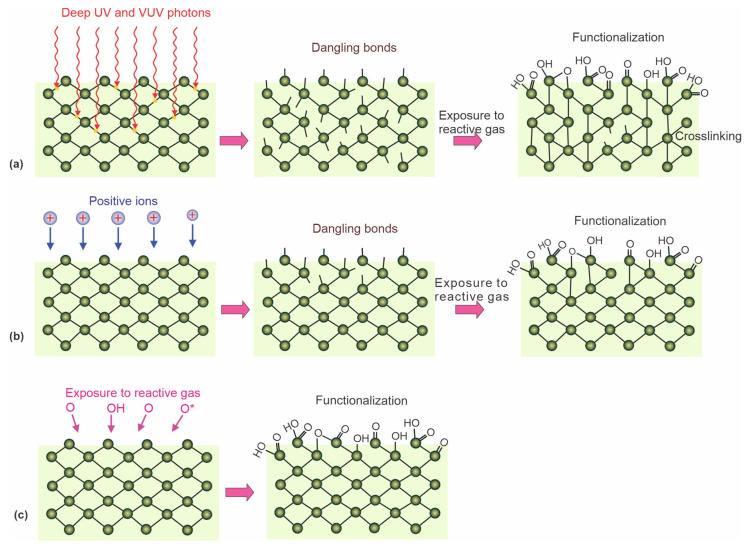
Illustration of the effects of various reactive plasma species: (**a**) energetic photons, (**b**) positively charged ions, (**c**) and neutral oxidizing plasma species on the surface finish of polymers.

The nonequilibrium gaseous plasma is a source of different reactive species interacting with the surface, causing its modification. In addition to atomic and molecular species (ionized, excited, or neutral radicals), plasma is also a source of extensive radiation, with the energy of some photons above the binding energy of atoms in polymer materials. In particular, radiation in a deep ultraviolet range (with photon energy above 6 eV) is of particular importance because such photons are capable of breaking bonds in polymers and thus causing the formation of dangling bonds. The resulting surface dangling bonds (free radicals) further react with atoms or molecules to form new functional groups [55,56,57]. The effect of deep ultraviolet radiation is illustrated in Figure 2a. The irradiation does not cause significant polymer heating, so it is a preferred method for hydrophilizing heat-sensitive polymers. The photons absorbed by the bulk polymer cause bond scission, so cross-linking is a common effect of polymer irradiation by plasma photons.

Energetic ions have similar effects as photons. The ions bombarding the surface cause bond breakage and phonon excitation because elastic collisions with atoms are more probable than bond scission. Dangling bonds formed in this way react with plasma species (Figure 2b). The advantage of using positively charged ions is a much lower penetration depth than photons. Therefore, the bond breakage is limited to a very thin surface film, often a few nm compared to that of photons, which is the order of µm. The disadvantage is significant surface heating because the entire ion energy (kinetic and potential) will cause heating due to elastic collisions with surface atoms and neutralization with surface electrons. 

The neutral reactive plasma species are useful for the hydrophilization of many types of polymers. They move randomly in the gas phase, and their temperature in nonequilibrium gaseous plasma is close to ambient temperature, so they will cause only marginal surface heating. Due to the marginal kinetic energy, they are unlikely to cause bond scission but will interact with the polymer surface by substituting hydrogen atoms with, for example, oxygen. This leads to the formation of various functional groups, such as hydroxyl, epoxy, carbonyl, and carboxyl. The effect of reactive oxygen species is illustrated in Figure 2c.

In addition to functionalization, another result of polymer treatment with plasma is etching. Etching may cause nanostructuring of the polymer surface and, thus, a significant increase in the surface area compared to the geometrical area. Such a rich surface morphology may be beneficial for improving the adhesion of coatings for at least two reasons: 1—a larger surface will provide more sites for negatively charged surface functional groups, and 2—the adhesion on a rough surface is mechanically favorable because of mechanical interlocking. 

Both effects of plasma treatment, i.e., functionalization and nanostructuring, have a strong influence on surface wettability, which is increased after plasma treatment. Good surface wettability of polymers is important for better spreading of the deposited coating over the sample surface, leading to a more uniform coating thickness. In the opposite case, if the wettability of polymers is not improved before applying a coating, the coating will be uneven and in the form of isolated islands. Therefore, plasma treatment has become an important technique for polymer surface modification, as it enables using different reactive gases and, thus, a different combination of final surface effects.

## 3. Overview of Plasma Methods for Chitosan Immobilization

This section reviews plasma methods of polymer treatment for better adhesion of chitosan coatings. The main contributions of different authors are presented, and a comparison of different treatment procedures and corresponding results is given. Many authors used a one-step method, i.e., plasma treatment followed by direct chitosan deposition [58,59,60,61,62,63,64,65,66,67,68,69,70]. Other authors preferred a two-step method using an additional intermediate layer [43,44,71,72,73,74,75,76,77,78,79,80]. In the latter case, plasma-treated polymers were first grafted with desired molecules, followed by chitosan deposition. The results are summarized in Table 1 and Table 2 for direct and indirect deposition, respectively. All important parameters, such as the type of material substrate, treatment procedure, and wettability, are presented as well. Various authors used different experimental setups, procedures, and substrate materials; therefore, the results are hardly comparable. The most important parameters that the authors reported were the type of substrate materials, the gas or gas mixtures, and the pressure and treatment time, as well as the type of discharge used for sustaining gaseous plasma, which will be presented in the following subsections. The thickness of the chitosan coating was rarely provided. The thickness of the chitosan coating may influence the lubricous properties of a chitosan coating. In some applications (i.e., urinary catheters [81], wound healing [82] and scaffolds for tissue engineering [83]), a chitosan gel-like structure is needed, which should be formed in contact with water. As shown by Vesel et al. [81], the contact angle of the chitosan coating was smaller for double-deposited coating compared to a single-layer coating.

## 4. Direct Deposition of Chitosan on Plasma-Treated Polymers

**Figure 3:**
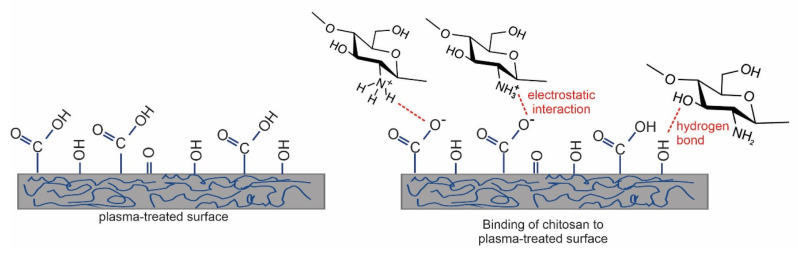
Different types of noncovalent interaction of chitosan with the plasma-treated surface [81].

As already mentioned, the straightforward method for forming functional groups on polymer surfaces is treatment with nonequilibrium gaseous plasma, which benefits from one or more effects illustrated in Figure 2. Negatively charged surface functional groups are responsible for stabilizing the chitosan layer through noncovalent interactions such as electrostatic interactions [86,87,88]. In particular, carboxyl functional groups may interact with ammonium functional groups of chitosan, which are transformed into protonated NH_3_^+^ groups in acidic solutions [89]. There may also be hydrogen bonding between OH groups on the plasma-treated surface and OH groups of chitosan [87,88,89]. These interactions are schematically presented in Figure 3.

Different plasma treatments were reported for surface functionalization of substrate materials using low-pressure oxygen or argon plasma treatment and atmospheric air plasma treatment (Table 1) [58,59,60,61,62,63,64,65,66,67,68,69,70]. In one case, nitrogen plasma was used because it has a positive effect on the biocompatibility of the surface; however, the substrate was first pretreated with air [69]. Therefore, it can be concluded that oxygen-containing plasmas should be used for the direct immobilization of chitosan. In the next two sections, the individual contributions of various authors are presented in more detail. For clarity, the first section deals with low-pressure plasma treatment, and the second addresses atmospheric pressure plasma treatments.

### 4.1. Examples of Low-Pressure Plasma Treatments for Direct Deposition of Chitosan

As early as 2002, Shin et al. [58] exposed linear low-density polyethylene (LLDPE) foils to oxygen plasma. Plasma was sustained in oxygen at the pressure of 0.13 Pa with an antenna coupled to a radiofrequency (RF) generator operating at the frequency of 13.56 MHz and the power of 200 W. The LLDPE foils were placed on an electrode powered by a DC-pulsed generator with a pulse duration of approximately 10 µs and a voltage of about 5 kV. The duty cycle was approximately 1 ms. The gaseous plasma sustained by the RF antenna was a source of ions, which were accelerated using the pulsed generator to hit the polymer samples. The ion kinetic energy was approximately 5 keV, and the samples received a dose of oxygen ions of approximately 1.7 × 10^18^ m^−2^. Such a dose caused the formation of nano topography as well as surface functionalization. The treatment with oxygen ions caused a drop in the WCA from the original value of 86° down to 6°, thus obtaining a practically superhydrophilic surface finish. The high-resolution XPS C1s spectra revealed the formation of mostly C–O bonds, followed by C=O and O–C=O. A chitosan film of a thickness of approximately 50 µm was deposited on the treated substrates. Such a chitosan film caused 10 times lower oxygen permeability than the nontreated LLDPE foils. The predominant plasma mechanism used by Shin et al. [58] is illustrated in Figure 2b. Excessive heating was avoided by pulsing the ion source. According to Table 1, Shin et al. [58] is the only author who reported an almost superhydrophilic surface finish of the substrate before chitosan deposition. Polyethylene will usually not become superhydrophilic upon treatment with O_2_ plasma [49], so it is worth explaining Shin’s method.

**Figure 4:**
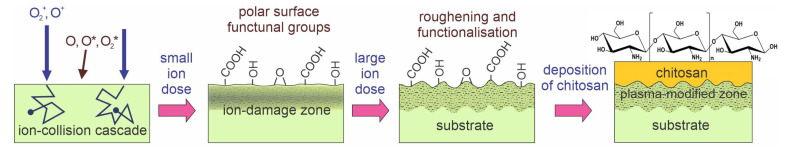
Evolution of surface morphology and polar groups upon treatment with low-pressure oxygen plasma and simultaneously bombarded with O_2_^+^ and O^+^ ions in pulses.

Oxygen ions with a kinetic energy of 5 keV not only cause bond scission and formation of dangling bonds, as illustrated in Figure 2b, but also the displacement of atoms in the surface film and sputtering, i.e., etching of the material surface by kinetic effects. These reactions cause a loss of the ion’s kinetic energy, so the oxygen ion is implanted after several collisions. The subsurface film thus contains some implanted oxygen that reacts with the surrounding atoms in the polymer and thus charges the material within a depth with a thickness equal to the penetration depth of 5 keV oxygen ions (several nm). The kinetic energy of ions is several orders of magnitude larger than the binding energy of atoms in the polymers, so the ions cause significant modifications of the surface film. The surface film with a thickness of several nm will have completely different properties compared to the bulk polyethylene, so there will be a mismatch in mechanical properties between the surface film and the bulk polymer [90]. As a result, the polymer will assume a nanostructured morphology [90]. Because Shin kept the samples in a chamber rich in O atoms, the surface was saturated with oxygen-containing functional groups during the ion bombardment. The combination of rich morphology and dense surface functional groups is the condition for the superhydrophilicity of polymers [91]. Such surface morphology evolution is typical for polymers treated with energy ions of the order of keV [90]. Shin’s method [58] is illustrated in Figure 4.

**Figure 5:**
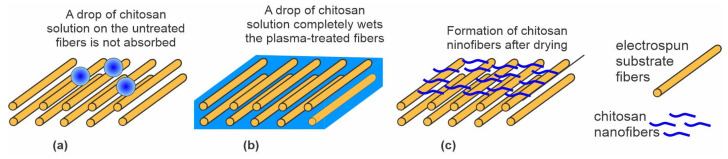
The effect of plasma treatment on the soaking properties of electrospun fiber cloth. (**a**) Droplets of chitosan solution remain on the untreated cloth surface after dipping into a chitosan solution; (**b**) plasma-treated cloth absorbs the solution within a few seconds of dipping; and (**c**) chitosan nanofibers are formed on the freeze-dried samples.

Jing et al. [59] also used low-pressure O_2_ plasma but without pulses of energetic ions. The authors treated polypropylene carbonate (PPC) microfibers prepared by electrospinning. The microfibers of an average diameter of 1.5 µm exhibited a rough surface with densely distributed cracks typically perpendicular to the length of the fibers. A possible application of these materials is as scaffolds in tissue engineering. The authors reported the O_2_ pressure during the plasma treatment to be as low as 0.04 Pa, achieved during continuous pumping and leakage of O_2_ at a flow rate of 50 sccm. If the reported pressure was correct and the samples were placed on the housing of the discharge chamber (not RF biased), the treatment involved a combination of effects illustrated in Figure 2a,c. This means that nonbiased samples are at a floating potential during plasma treatment, so the kinetic energy of positive ions is marginal, at approximately 10 eV. The treatment increased both C–OH and O=C–OH functional groups. The plasma-treated microfibers exhibited moderately hydrophilic properties. The WCA of the plasma-treated substrates was decreased to 51° after 5 s and to an immeasurably low value after 10 s. The water droplet was, therefore, soaked by the microfiber network. Oppositely, the WCA of the untreated sample remained at approximately 120° and was dried instead of being soaked (Figure 5a). The plasma-treated surface of PPC microfibers was suitable for grafting chitosan. Chitosan powder was dissolved in slightly acidic water, and the scaffolds were immersed in the chitosan solution for 2 min at room temperature. The soaked microfibers (Figure 5b) were then placed into liquid nitrogen, and deep-frozen samples were mounted in a vacuum chamber for drying. Using an innovative technique, the authors reported the formation of chitosan nanofibers with a diameter of around 250 nm on the surface of the microfibers (Figure 5c). The wettability of substrates grafted with chitosan nanofibers was significantly improved compared to plasma-treated microfibers. The soaking time was reduced by a factor of two or so. Therefore, Jing et al. [59] provided a procedure for optimal hydrophilicity of a chitosan coating: freeze drying of substrates soaked in a chitosan solution. The method used by Jing et al. [59] is illustrated in Figure 5.

Suganya et al. [60] treated polystyrene (PS) with a low-pressure gaseous plasma sustained in argon, oxygen, and air at the pressure of 2 Pa using a DC discharge operating at a voltage of 300 V and power of 100 W. The distance between the electrodes was 6 cm, so the product of electrode distance and pressure was only 0.16 cm × mbar. This is below the Paschen minimum, which defines a breakdown voltage to start a discharge depending on pressure and distance between the electrodes [92]. Therefore, it is surprising that the discharge was sustained at such a low voltage. PS is electrically an insulator; thus, its surface was at the floating potential, resulting in ion kinetic energy upon impinging the polymer surface of 10 eV. DC discharges are not famous as extensive deep UV sources; therefore, the surface kinetics for this type of discharge is best illustrated by Figure 2c. The residual atmosphere in the vacuum chamber during Ar plasma treatment was probably the source of oxidative species (OH radicals and O atoms). Plasma-treated foils were immediately dipped into the chitosan solution to avoid polymer hydrophobic recovery. The unbonded chitosan was removed by rinsing the foils with distilled water. The WCA decreased gradually with a plasma treatment time of up to 15 min and stabilized at approximately 40°. A rather large WCA for plasma-treated samples could be explained by a very small flux of reactive plasma species or thermal effects. XPS revealed 20 at.% of oxygen and also 3 at.% nitrogen after O_2_ plasma treatment. The origin of nitrogen is unclear and unlikely to be due to the leakage of the vacuum system because the working pressure was as low as 2 Pa. SEM images clearly revealed that the chitosan was not bonded in the form of a thin film but rather nanoparticles of spherical shape and a typical diameter of a few 100 nm. Polystyrene Petri dishes with a chitosan coating were used to study the preservation of grapes, and the results were impressive since the weight loss decreased from 35% for grapes placed into untreated dishes to 5% for dishes coated at optimal conditions, i.e., O_2_ plasma treatment for 15 min, followed by deposition of chitosan. It is noteworthy that other authors reported much different surface finish of PS upon plasma conditions, i.e., rapid hydrophilization upon low-pressure O_2_ plasma treatment [93,94,95,96].

In 2019, Glaser et al. [61] treated polyethylene (PE) and polypropylene (PP) foils with neutral reactive oxygen species (predominantly O-atoms in the ground state) from the flowing afterglow of O_2_ plasma sustained at the pressure of 50 Pa by a microwave (MW) discharge in the surfatron mode. The treatment is illustrated in Figure 2c. They dispersed chitosan in an acidic aqueous solution and formed particles of a typical linear dimension just below 1 µm. The mass of the chitosan microparticles distributed on the polymer surfaces was of the order of 0.1 g/cm^2^, so the equivalent thickness of the chitosan film was roughly 1 mm if the reported mass was correct. Plasma treatment caused the formation of various oxygen functional groups, and the XPS O/C ratio of treated foils was approximately 0.16. A huge difference in O/C was reported after incubation with chitosan: 0.56 for plasma-treated and 0.10 for the untreated foils. The WCA value of untreated foils was 109° and dropped to approximately 30° after plasma treatment. This is similar to the minimal WCA reported for these two polymers [49]. The WCA values of samples coated with chitosan were approximately 90 and 43° for the untreated and treated samples, respectively. The difference in WCA may be explained by the formation of a rather uniform chitosan film on plasma-treated samples, while the coating on untreated samples was either full of cracks or consisted of nonevenly distributed chitosan particles because the mass gain was comparable for the untreated and plasma-treated samples. Since samples were prepared for food packaging application, the authors also measured the oxygen permeability of the chitosan-coated foils. The measured permeability for oxygen was 5–10 times lower compared to untreated foils coated with chitosan. The results demonstrated the role of plasma pretreatment in the uniformity of chitosan film. The polyelectrolyte titration experiments showed 10 times larger chitosan desorption for untreated samples compared to plasma-treated samples. 

Recently, Vesel et al. [81] treated urinary catheters made of Meliflex XP polyolefin-based compounds in a low-pressure RF hydrogen plasma, which is an extensive source of UV radiation. Treatment with hydrogen plasma was followed by a second treatment using oxygen plasma. Such a combination of treatment procedures obtained better hydrophilicity than only oxygen plasma treatment. UV photons from hydrogen plasma were responsible for creating reactive radicals (as shown in Figure 2c). Upon further exposure to oxygen plasma, enhanced formation of oxygen functional groups was observed. Plasma-treated catheters were incubated in chitosan solution for 20 min and then dried to deposit a single-layer coating. In another example, single-layer coated catheters were incubated in chitosan solution to deposit another coating. The wettability of the two-layered coating was better than the single one. 

Komoto et al. [62] reported chitosan-coated poly(L-lactic acid)–PLA fibers for suture threads. They used a low-pressure RF discharge operating at the frequency of 100 kHz and discharge power of 100 W to sustain O_2_ plasma. The treatment times varied between 30 and 1800 s. Details of the plasma reactor and discharge parameters were not disclosed, but it seems that the predominant mechanism of Komoto’s method was that which is illustrated in Figure 2c. The plasma-treated samples were immediately immersed in chitosan solution for a minute. The solution was then freeze dried to remove excess acetic acid and water. The chitosan–acetate salt was then dissolved in pure water. After depositing chitosan salt, the samples were rinsed with distilled water to obtain a single chitosan layer. A layer of sodium alginate was then deposited similarly. The procedure was repeated several times to obtain multilayered structures on the PLA fibers. Up to 15 chitosan layers were prepared. The thickness of the chitosan varied between 30 and 100 nm. The nitrogen concentration versus the number of chitosan layers was monitored by XPS. The N/C ratio was approximately 0.27 for 1 layer and 0.04 for 3 layers of chitosan, and it remained at this value for further layers. Based on XPS results, the authors conclude that the thickness of the chitosan coating on the PLA fibers increased linearly with the increasing number of layers. The samples degraded marginally even after several months of storage in PBS solution. The coating was, therefore, a multilayer of chitosan and sodium alginate. Freeze drying was a proper technique, but Komoto did not mention the formation of fibrous chitosan, as reported by Jing et al., who also used the freeze-drying method [59].

Similar to Komoto [62], who prepared multiple layers of chitosan, Arkhangelskiy et al. [42] also prepared a multilayer structure but using a different approach. Arkhangelskiy et al. [42] performed plasma-assisted deposition of chitosan using a plasma torch directed toward the sample surface. In this case, chitosan aerosols were fed into plasma. Aerosols of chitosan were made by ultrasonication of chitosan solution, which was injected into a plasma torch. Ar gas was used as a carrier gas to deliver aerosol precursors to the sample. Nitrogen gas was also used. It was used as a cooling gas. This unique technique enabled layer-by-layer deposition of chitosan. If using an additional PDMS mask, the technique enabled even the formation of patterned surfaces.

### 4.2. Examples of Atmospheric Pressure Plasma Treatments for Direct Deposition of Chitosan

Apart from low-pressure plasmas, the plasmas sustained at atmospheric pressure are also useful for modifying polymer surface properties for better adhesion of chitosan. Nawalakhe et al. [63] disclosed a method for depositing chitosan nanofibres on cotton gauze. The gauze was subjected to He plasma sustained at atmospheric pressure with a dielectric barrier discharge (DBD) powered with a 1.37 kHz generator at a peak voltage of approximately 7 kV. Plasma was also sustained in a mixture of He with 1 vol.% O_2_, but the wettability was better after treating it with pure He plasma. This rather unexpected result is explained by suppressing the deep ultraviolet (UV) radiation upon mixing He with O_2_. Pure He atmospheric pressure plasma is an extensive radiation source from the relaxation of He_2_* excimers. The photons cause bond scission, as illustrated in Figure 2a. When O_2_ is added, the He metastables are quenched, so the radiation is suppressed significantly [97]. The chitosan nanofibers were deposited by electrospinning of chitosan dissolved in trifluoroacetic acid (TFA). Long chitosan nanowires of a diameter of 250 nm were deposited when using a 7% chitosan solution. The He plasma pretreatment enabled four times better adhesion between the chitosan nanofiber mats and the supporting cotton fabrics. In fact, the difference between untreated and plasma-treated samples was observed with the naked eye after the Gelbo testing. The coating was bactericidal for *E. coli* and *B. cereus*. The authors concluded that the methods might enable faster wound healing.

**Figure 6:**
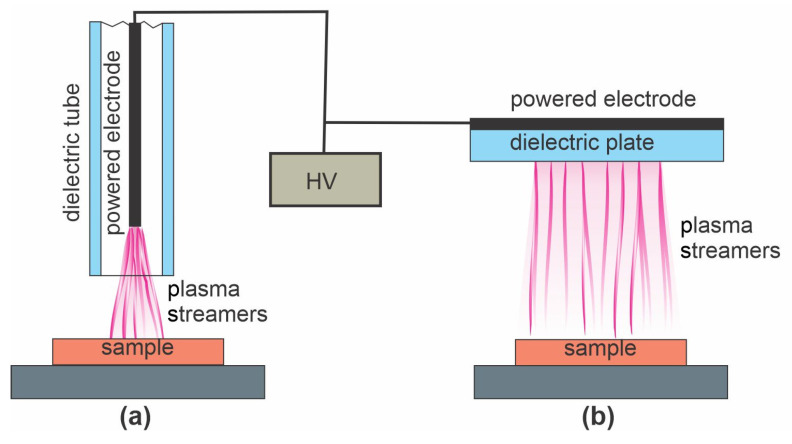
(**a**) The atmospheric plasma jet and (**b**) plasma sustained by dielectric barrier discharge (DBD) discharge with a high-voltage generator at a frequency below 10 kHz. The sample holder may or may not be grounded.

Lei et al. [64] treated polymer foils with a PP surface film with atmospheric pressure plasma sustained in the air with a DBD discharge. The plasma treatment time was 180 s, which resulted in the WCA dropping from 92 to 68°. PP is renowned for its poor wettability after plasma treatment, so this result is not surprising [49]. After the plasma treatment, the samples were immersed in an acidic aqueous chitosan solution and dried to form a thin film of solid chitosan. The chitosan was slowly released upon immersion into various liquids, but the antibacterial properties of the samples were retained, so the authors found their method useful for food packaging. The major reactants in air plasma at atmospheric pressure are atomic and molecular radicals, so Lei’s [64] approach is best illustrated by Figure 2c. The ion kinetic energy is always marginal because of numerous elastic collisions with neutral particles at atmospheric pressure. The plasma methods using the atmospheric plasma jet and DBD discharge, both sustained at a frequency below approximately 10 kHz, are illustrated in Figure 6a–b, respectively.

Ren et al. [65] used an atmospheric pressure plasma to activate ultrahigh-molecular-weight PE fibers before chitosan deposition. The chitosan was dissolved in 2% *w/v* acetic acid aqueous solution to obtain a 0.7% *w/v* concentration of chitosan solution. Plasma was sustained with a DBD discharge, as illustrated in Figure 6b, in a mixture of Ar and 10 vol.% O_2_. The treatment times varied between 40 and 140 s. The WCA was measured for the untreated fibers and those coated with chitosan after various plasma treatment times. The WCA on samples covered with chitosan decreased almost linearly with the treatment time up to 100 s. The decreased wettability was explained by the coverage with chitosan. The untreated samples dipped into a chitosan solution exhibited poor wettability because chitosan did not remain on the fibers’ surface. When increasing the plasma treatment time, the chitosan concentration increased, and the WCA decreased. The high-resolution XPS C1s peaks of samples treated with plasma for 100 s and then dipped into the chitosan solution were almost identical to pure chitosan, so it seems that the chitosan film was compact and of a thickness above the XPS probing depth, which is approximately 10 nm [98]. The plasma method used by Ren et al. [65] is best illustrated in Figure 2c. A rather large concentration of O_2_ in Ar caused the quenching of Ar* metastables and a rather low electron temperature, and both suppressed deep UV radiation and facilitated the formation of O atoms, ozone, and O_2_* metastables, which interacted chemically with the polymer surface.

In a recent paper, Carette et al. [66] reported an improved interface between polyesters and chitosan. The polyester substrates were treated with an Ar plasma jet sustained at atmospheric pressure with an MW discharge with a power of 30 W. The plasma kinetics of jets powered with MW sources are completely different from jets sustained with low-frequency high-voltage sources, so Figure 6a is void for this case. The continuous (not in streamers) plasma plume at elevated temperature was scanned over the substrate surface. The WCA on the untreated samples was approximately 85° and dropped to approximately 55° after the treatment. The high-resolution XPS spectra revealed a significant increase in the peak at the binding energy of 289 eV assigned to O=C–O and C=O. The O–C=O peak was more significant than C–C, and the C–O peak was as large as C–C. Such an extensive functionalization should cause a much lower WCA [99]. The discrepancy between the XPS and WCA results, as reported by Carette et al. [66], could be explained by thermal effects. The hot plasma sustained by MW at atmospheric pressure causes significant polymer heating upon treatment. The polar groups could be migrated inside the polyester, so the wettability (depending solely on the surface functional groups) remained moderate. Still, such moderate hydrophilicity enabled a good adhesion of chitosan on plasma-treated substrates upon dip casting, which was determined by Fourier transformed infrared spectroscopy (FTIR). FTIR spectra of untreated samples dipped into the chitosan solution were similar to the spectra of the substrate, while the samples pretreated with Ar plasma and dipped into the chitosan solution revealed a broad peak typical for chitosan. 

In another paper, Carette et al. [84] reported the same results as in [66], but they added AFM images of samples treated with Ar plasma. The plasma treatment caused nanostructured surface morphology typical for plasma treatment of many polymers. The AFM images showed conical morphological features of a few 100 nm lateral dimensions and a height of approximately 50 nm. Such a rough surface finish, together with functionalization, enabled the adhesion of a rather uniform chitosan coating. The surface finish was, therefore, similar to the one illustrated in Figure 4, except that the surface was not rich in polar groups, probably because of the thermal effects. Good stability of the chitosan film was reported, so the method was found to be useful for coating polymers to achieve antibacterial properties. The properties were tested for both *E. coli* and *S. aureus*.

## 5. Chitosan Deposition on Plasma-Treated Polymers with an Intermediate Layer

**Figure 7:**
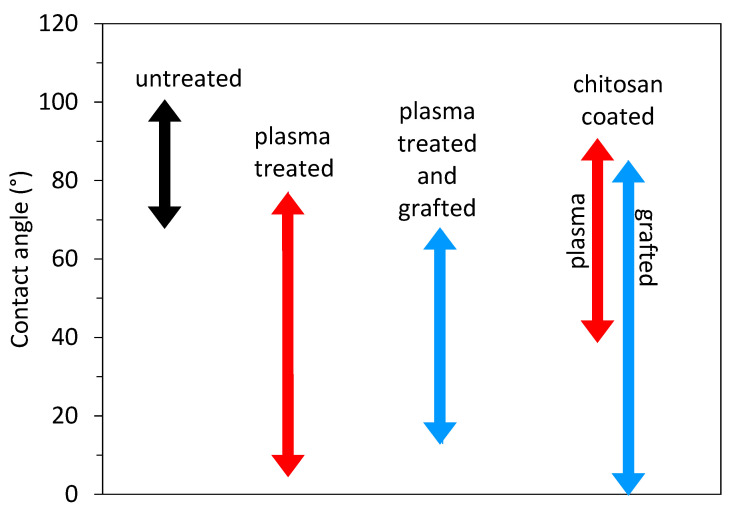
Reported wettability of the untreated substrate materials (black arrow), plasma-treated substrates (red), grafted substrates (blue), and chitosan-coated substrate (red for plasma-treated substrates and blue for grafted substrates).

As shown in the previous section, the single-step method, i.e., plasma treatment followed by dipping in a chitosan solution, enables the formation of a thin chitosan film. Although different authors obtained different hydrophilicity of plasma-treated polymer substrates ranging from almost superhydrophilic surface with WCA of 5° to moderate hydrophobic surface with WCA of 68° (for a polymer foil) or even 92° (for polymer microfibers), chitosan was found on all surfaces. This means that other factors or their combination, i.e., concentration and type of surface functional groups and roughness, are more important for the immobilization of chitosan than just excellent wettability, which is important for the uniform spreading of the chitosan solution over the substrate surface. This is further examined by a comparison of a direct single-step method (presented in Table 1) with a more complex indirect two-step approach (summarized in Table 2). This approach uses an intermediate layer deposited on plasma-treated substrates using polymer grafting (usually acrylic acid). Additionally, in this case, the reported hydrophilicity of the grafted layer ranged from very hydrophilic to moderately hydrophobic. Figure 7 shows the range of the reported wettability of the untreated substrate material, plasma-treated substrate, and grafted substrate. We can see that the additional grafted layer does not provide better wettability than the plasma-treated one, which means that moderate surface hydrophilicity is sufficient for successfully immobilizing chitosan if the right type of surface functional group is present. However, the uniformity of such coating is still questionable. Authors have rarely reported the adhesion force. The adhesion of chitosan and its uniformity on surfaces with moderate hydrophilicity may not be the best.

Substrates coated with chitosan are often used in medical applications, where they have to form a gel-like surface when coming into contact with water to ensure high surface lubricity. Therefore, it is also interesting to examine the wettability of the chitosan-coated substrates. This information is also presented in Figure 7. The reported wettability of the chitosan varied from superhydrophilic to hydrophobic, which is difficult to explain. Probably, the coating was not uniformly distributed over the surface, and there might be some uncovered areas, or it can also be a consequence of different surface roughness since some authors reported nanoparticles of even chitosan fibers [59,60]. 

**Figure 8:**
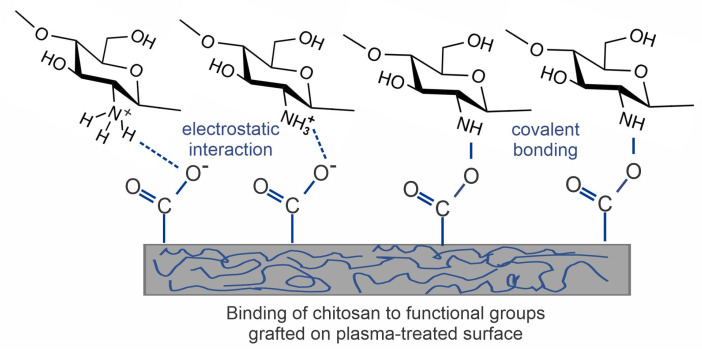
Covalent and noncovalent interaction of chitosan with a plasma-treated surface.

As already mentioned, the force of adhesion of a chitosan layer is usually not reported. The force might not be optimal; therefore, a more complex indirect approach (as summarized in Table 2) is advisable because it enables the covalent bonding of chitosan, as illustrated in Figure 8. This approach involves depositing an intermediate layer of acrylic acid to obtain carboxylic groups. Acrylic acid is sometimes additionally combined with PEG. Covalent binding of chitosan to the intermediate layer is performed by using different coupling agents. The authors mostly used an EDC coupling agent, often combined with NHS [43,44,68,71,72,75]. Other coupling agents, such as DECH and EDAC, were rarely used [73,77,79,80]. The coupling agents help to induce cross-linking of amines from chitosan with carboxylic groups by promoting covalent bond formation [44]. This is a reason for using acrylic acid as an intermediate layer. 

However, Paslaru et al. [68] also tested the application of coupling agents directly on the plasma-treated substrate without depositing an intermediate grafted layer. Paslaru et al. performed corona plasma treatment of PE surfaces, followed by immediate chemical activation of the plasma-treated surface with EDC and NHS coupling agents to bind chitosan. They found that the grafted chitosan layer was more stable than the physically adsorbed one. In another recent paper, Stoleru et al. [100] used nitrogen plasma treatment to activate PLA films. Chitosan coating was covalently bound using the activation of hydroxylic groups with an EDC coupling agent. This procedure also enabled the deposition of chitosan coatings with embedded clove and argan oils. 

Although it seems that this procedure (plasma followed by direct application of coupling agents) can also provide good results, all other authors decided to deposit an intermediate layer on the plasma-treated surface before further deposition of chitosan. Below are some examples of the two- or multiple-step methods reported by various authors.

### 5.1. Examples of Low-Pressure Plasma Pretreatment for Indirect Deposition of Chitosan via an Intermediate Layer

**Figure 9:**
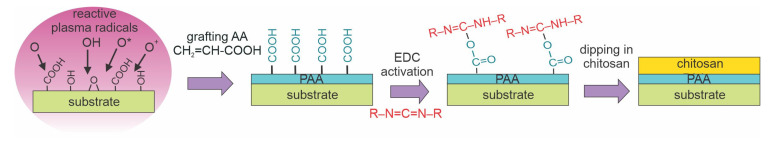
A method for immobilizing a thin chitosan film on plasma-treated samples grafted with an intermediate layer. Oxygen plasma-treated substrates are grafted with acrylic acid to form carboxyl groups, activated with carbodiimide reagent, and dipped into an acidic aqueous chitosan solution.

Tyan et al. [71] treated nonwoven PP fabrics with O_2_ plasma sustained by an MW discharge. The estimated density of positively charged oxygen ions was between 1 × 10^15^ and 1 × 10^16^ m^−3^ at the pressure of 33 Pa. The plasma treatment time of 10 s enabled hydrophilization of otherwise hydrophobic substrates. XPS analyses showed a large oxygen concentration on the surface of plasma-treated fabrics with an O/C ratio of 0.45. The concentration of C–O bonds was the largest, but the concentrations of C=O and O–C=O groups were almost identical. A chitosan film was not deposited directly on the plasma-treated fabrics, but the substrates were soaked with an aqueous solution of acrylic acid (AA) to form an intermediate layer of polyacrylic acid (PAA). The fabrics were then soaked in 1-ethyl-3-(3-dimethylaminopropyl) carbodiimide (EDC) to activate the O=C–OH groups on the PAA surface. After this step, the samples were immersed in an acidic aqueous chitosan solution and carefully dried at 4 °C. The substrates grafted with PAA were hydrophilic and capable of regaining moisture. The samples prepared with Tyan’s method could absorb approximately six times more water than the non-Tyan-treated samples, which made the modified PP fabrics feasible as an intensive absorbent and coupling support to promote the immobilization of chitosan via amide bonds. The treatment enabled improved hemocompatibility of the PP fabrics. Tyan’s procedure is illustrated in Figure 9. This figure shows oxygen plasma-treated substrates grafted with acrylic acid to form carboxyl groups, then activated with EDC, and finally dipped into an acidic aqueous solution of chitosan to form a layer of well-immobilized chitosan.

Lin et al. [72] immobilized chitosan on plasma-treated polyurethane (PU) membranes. O_2_ plasma was sustained in a metallic chamber and powered by a capacitively coupled RF discharge. The treatment times were between 20 and 150 s. The evolution of surface peroxide groups was measured with titration. The maximum amount of the peroxide groups of 150 nmol/cm^2^ was reported for the treatment time of 90 s. The plasma-treated polyurethane membranes were grafted with acrylic acid, and the amount of the grafted reagent followed the concentration of peroxide groups. An aqueous chitosan solution was then grafted on the film of grafted PAA. Chitosan was immobilized similarly to that illustrated in Figure 9. The hemocompatibility of the polyurethane membranes coated with chitosan was significantly improved compared to nontreated membranes. 

Zhu et al. [73] reported improved hemocompatibility of polyethylene terephthalate foils (PET) treated with Ar plasma. The PET samples were placed in a low-pressure plasma reactor otherwise used for reactive ion etching of various materials. Plasma was sustained with a capacitively coupled RF plasma at 200 W. The ultimate pressure in the reactor was approximately 3 Pa, and Ar was leaked during continuous pumping at the rate of 20 sccm. The samples were grafted with acrylic acid right after the plasma treatment. The acrylic acid was polymerized by UV radiation using a mercury lamp at 100 W/m^2^. The samples were then immersed in an aqueous solution of chitosan, dried, rinsed with 1% acetic acid, and then rinsed for a day with high-purity water to remove the weakly bonded chitosan. The topmost layer was, therefore, covalently bonded chitosan with a rather high wettability since the WCA was between 15 and 20°. The procedure was similar to that illustrated in Figure 9, except that [71] and [72] did not use UV curing and a (3-dimethylaminopropyl)-3-ethyl-carbodiimide hydrochloride (DECH) coupling agent. The platelet-adhesive and protein-adsorptive resistance were greatly improved compared to untreated PET foils. 

**Figure 10:**
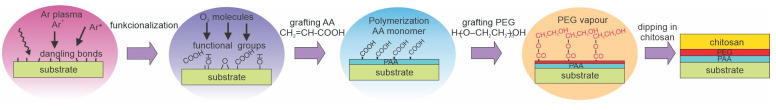
A method for immobilizing a thin chitosan film using the following steps: formation of dangling bonds by Ar plasma, interaction of dangling bonds with oxygen, plasma polymerization of a layer resembling cross-linked polyacrylic acid (PLA), exposure to polyethylene glycol (PEG) vapors, and dipping in chitosan solution.

Pandiyaraj et al. [74] treated low-density polyethylene (LDPE) with Ar plasma. Plasma was sustained with a DC discharge, and the polymer foils were placed on the anode to keep them close to the floating potential. The DC discharge does not enable bombarding the insulating materials with energetic ions, so the surface kinetics were probably similar to those illustrated in Figure 2a. Plasma treatment lasted for 5 min. After turning off the Ar plasma, O_2_ was introduced into the plasma reactor. The authors explained surface hydrophilization as being due to the formation of dangling bonds upon treatment with Ar plasma. The bonds then reacted with oxygen to form functional groups, as illustrated in Figure 2b. Such a surface finish was useful for the next step, the deposition of a PAA film through plasma polymerization of acrylic acid. The samples were then grafted with polyethylene glycol (PEG) using the same reactor. A layer of PEG-resembling film acted as a spacer between the substrate and a layer of chitosan, which was deposited from an aqueous solution. This method is schematically illustrated in Figure 10. The water contact angle of untreated LDPE was 95° and dropped to 57° after plasma treatment. The WCA on the samples coated with a thin chitosan film was as low as 16° and remained low upon prolonged storage at ambient conditions. The WCA was 20° after 15 days. Samples were probed by AFM. Initial roughness was very low at an R_a_ of approximately 2 nm and increased to approximately 15 nm after Ar plasma treatment. It smoothened due to the deposition of the PAA film, increased to 15 nm after polymerization of PEG, and finally stabilized at 10 nm after chitosan immobilization. The authors reported excellent hemocompatibility because the platelets did not adhere to the chitosan-coated surfaces. 

**Figure 11:**
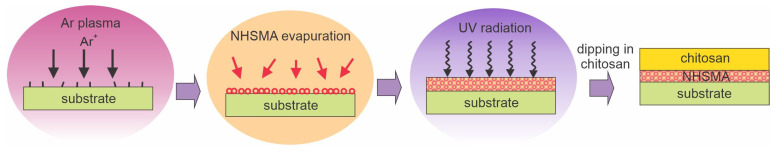
A method for immobilizing a chitosan film using the following steps: formation of dangling bonds by bombardment with Ar ions, evaporation of NHSMA, cross-linking of the deposited film by UV radiation, and dipping in an acidic aqueous solution of chitosan.

Tardajos et al. [76] reported the antibacterial properties of a chitosan film deposited on plasma-activated polycaprolactone (PCL) electrospun fibers using methacrylic acid N-hydroxysuccinimide (NHSMA) as an intermediate grafted layer. Plasma was sustained in Ar using a capacitively coupled discharge of 100 W. The plasma effect is best illustrated in Figure 2b. The best plasma treatment time was found to be 30 s, which enabled further grafting of the NHSMA and cross-linking using a 30 min exposure to UV radiation. The fibers grafted with cross-linked NHSMA were then incubated in a 0.5–1% chitosan solution in 0.1 M MES buffer at pH 5. The procedure enabled the immobilization of a thin chitosan film, which was qualitatively confirmed by coloration. XPS spectra indicated a composition similar to chitosan so that one can deduce a good coverage of the fibers. The chitosan coating enabled bacteriostatic properties. The relative growth measured a day after incubation was approximately 35% for *S. aureus* and 20% for *S. Epidermidis*. The Tardajos method [76] is illustrated in Figure 11.

**Figure 12:**
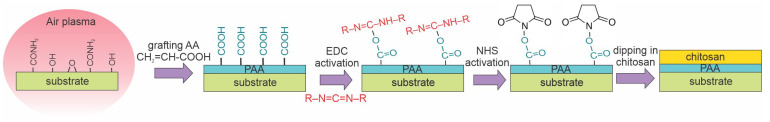
A method for immobilizing a chitosan film using the following steps: formation of functional groups by exposure to air plasma, grafting with acrylic acid, optional application of carbodiimide chemical coupling, and dipping in acidic aqueous solution of chitosan.

Recently, Karakurt et al. [44] reported the antibacterial properties of PLA films coated with a chitosan layer. Both a direct coating of the substrate and the usage of carbodiimide as an intermediate layer were reported. The samples were treated with low-pressure air plasma sustained with a capacitively coupled RF discharge at a power of 50 W. The air pressure was 60 Pa, and the treatment time was 60 s. PLA samples were placed on grounded metallic holders, and the discharge was sustained in an asymmetric configuration, so Figure 2c best illustrates the surface kinetics. The samples were incubated with acrylic acid to provide binding sites for the coating. Chitosan was covalently bonded to the substrates by dipping them into a complex chitosan-containing solution. The chitosan was adsorbed onto the pretreated substrates in the form of precipitates of the typical lateral dimension of several µm, as revealed in SEM images. They were 82° for untreated substrates and 46° after treatment with gaseous plasma. The WCA on samples coated with chitosan was approximately 65°. The plasma treatment caused the formation of several functional groups containing oxygen and/or nitrogen, as revealed by XPS. The nitrogen concentration in the surface film was approximately 3 at.%. The biocompatibility of chitosan-coated substrates increased significantly, and so did the antibacterial activity. Karakurt’s method [44] is illustrated in Figure 12.

**Figure 13:**
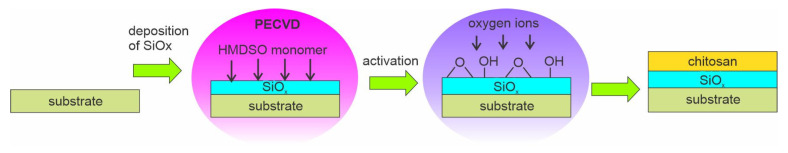
A method for immobilization of a chitosan film using SiO_x_ intermediate layer.

Opposite from the examples shown above, where the authors mostly used acrylic acid for grafting COOH groups, Li et al. [85] prepared multilayered PLA/SiO_x_/chitosan samples. First, the authors used the plasma-enhanced chemical vapor deposition (PECVD) method to deposit an intermediate layer of SiO_x_ on the PLA surface. A SiO_x_ layer was deposited using HDMSO monomer and O_2_ gas. After that, the SiO_x_ layer was posttreated using oxygen bombardment for 30 or 60 s. The procedure is schematically presented in Figure 13. Such substrates were ready for further deposition of the chitosan layer using a bar coater. After drying, the thickness of the chitosan coating was 40 μm.

### 5.2. Atmospheric Pressure Plasma Pretreatments for Indirect Deposition of Chitosan via an Intermediate Layer

**Figure 14:**
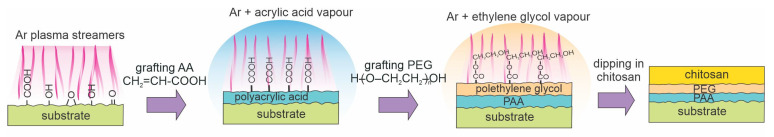
A method for immobilizing chitosan using two different intermediate layers, both prepared by plasma polymerization at atmospheric pressure, using Ar with an admixture of acrylic acid or ethylene glycol vapors.

As shown in the previous section, low-pressure plasmas can facilitate acrylic acid grafting. This can also be achieved by using atmospheric plasmas, which are presented in this section. In 2018, Pandiyaraj et al. [75] prepared polypropylene (PP) substrates using a combination of different coatings, but the processing parameters differed significantly from [74], which is presented in Figure 10. The plasma activation of PP samples was performed in Ar at atmospheric pressure using an AC high-impedance discharge at 50 Hz and voltage as large as 14 kV. Plasma was sustained in the streamer mode at conditions similar to that illustrated in Figure 6b. Plasma treatment caused the formation of dangling bonds and roughening of the substrates (Figure 2b). Hydrophilization was obtained upon exposure of Ar plasma-treated samples to oxygen, as illustrated in Figure 14. A plasma polymer layer was deposited in the reactor filled with Ar at atmospheric pressure using acrylic acid as a monomer. A PEG-like coating was also deposited using plasma polymerization. As mentioned earlier, plasma polymerization causes the formation of highly cross-linked films of a polymer rich in the precursors’ groups. Dipping in an aqueous solution enabled the immobilization of a very thin chitosan film. The Ar plasma treatment of PP enabled a significant concentration of hydroxyl groups (21%), and a small peak attributed to C=O or O–C–O bonds (4%) was also observed by XPS. The WCA decreased after each treatment. It was 92° for the untreated PP sample, 65° after Ar plasma, 44° after depositing PAA film, 20° after depositing PEG-like film, and 16° after depositing chitosan. Marginal hydrophobic recovery of chitosan-coated samples was observed because the WCA increased to approximately 30° after storage for a month at ambient conditions. The hemocompatibility of the samples was tested. The samples were incubated with blood, the number of adsorbed blood platelets dropped by an order of magnitude, and the amount of adsorbed blood proteins was roughly halved, which proves the enhanced hemocompatibility properties of the samples. The procedure for the sample preparation is illustrated in Figure 14.

The same group as [75] reported a modified method in 2019 [78], in which they treated low-density polyethylene (LDPE) with a plasma sustained in organic gases at atmospheric pressure using a DBD discharge. The LDPE pretreatment was performed in a simple plasma reactor with Ar plasma. The discharge was powered with a 50 Hz AC generator at a voltage as large as 14 kV. The precursors for polymer film deposition using plasma polymerization were acrylic acid and PEG vapors. Plasma polymerization lasted 5 min. Chitosan was dissolved in an acetic aqueous solution and deposited by dipping the plasma-treated foils. Excessive chitosan was washed away, followed by drying. The WCA of the untreated LDPE was approximately 95°, and it gradually decreased when increasing plasma polymerization time down to approximately 15° after 5 min. The WCA for samples coated with chitosan was only a few degrees, and the superhydrophilic surface finish persisted for two weeks or more when stored in air at ambient conditions. The surface roughness also increased with an increasing plasma polymerization time up to approximately R_a_ = 10 nm. The chitosan coating caused a further increase up to 25 nm. This method is one of the few that enabled the superhydrophilic surface finish of samples with an uppermost chitosan layer. The method is similar to that illustrated in Figure 14.

**Figure 15:**
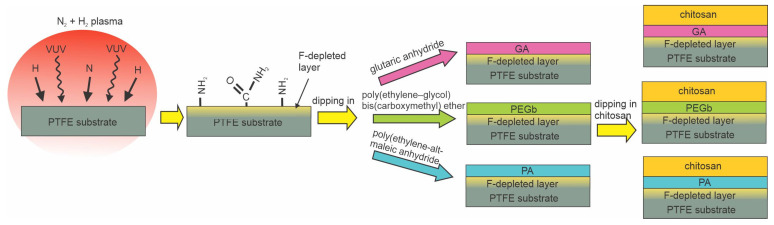
Immobilization of chitosan on polytetrafluorethylene.

DBD discharge was also used by Vaz et al. [77]. They reported the antibacterial properties of polytetrafluorethylene (PTFE) substrates coated with a chitosan layer using various intermediate layers. The PTFE samples were exposed to plasma sustained in a mixture of 95 vol.% N_2_ and 5 vol.% H_2_ at atmospheric pressure. The voltage of 10 kV at the frequency of 3 Hz enabled plasma in streamers rather than a continuum flux of reactive species, as illustrated in Figure 6b. The plasma treatment caused fluorine depletion from the PTFE surface, and XPS analyses showed approximately 5 at.% of nitrogen. The partial substitution of the fluorine with amino groups is probably due to bond scission upon irradiation with vacuum ultraviolet radiation (VUV) and the interaction of the dangling bonds with NH_2_ radicals from gaseous plasma [101]. The presence of amino groups was proved by chemical titration. Amino groups formed by plasma treatment served as anchor sites for the further binding of three different spacer molecules, glutaric anhydride (GA), polyethylene–glycol biscarboxymethyl ether (PEGb), and polyethylene-alt-maleic anhydride (PA). Chitosan was then grafted onto these spacer molecules. The layer of immobilized chitosan enabled the antibacterial properties for *X. fastidiosa* of all samples, but the best results (only a few % survival compared to control samples) were observed when polyethylene-alt-maleic anhydride was used as a spacer. The method is illustrated in Figure 15. Compared to other reports, the key innovative step is the substitution of fluorine in the surface film of PTFE with amino groups instead of oxygen groups and the immobilization of chitosan using different spacers.

**Figure 16:**
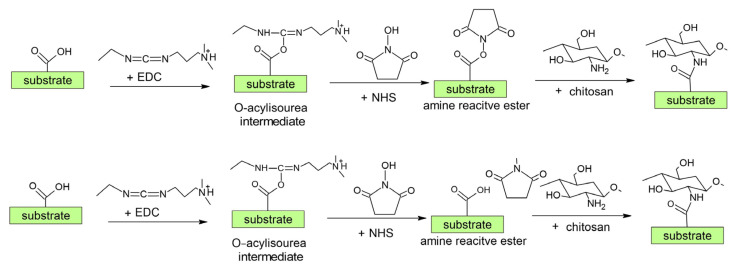
Scheme of the most commonly used procedure for the chemical coupling of chitosan (see Figure 12 also).

According to the literature review above (see also Table 2 and Figure 9 and Figure 12), we can conclude that at present, the most commonly used synthetic chemistry for coupling chitosan with a polymer is carbodiimide chemistry using EDC/NHS chemical agents [35,102]. The surface carboxylic acid group (from plasma treatment and polyacrylic acid grafting) is activated by the EDC reagent to produce an intermediate unstable group (Figure 16). This group is then transformed into a more stable NHS-activated carboxylic acid group, which is further coupled with the chitosan amino group [35,102]. It should be mentioned that the reaction selectivity of EDC could be questionable since it can react with the amino and hydroxyl groups. Moreover, because chitosan is a macromolecule, its steric hindrance may influence the coupling rate of chitosan [35]. This can lead to a lower and insufficient amount of chitosan on the surface.

## 6. Conclusions

A variety of methods useful for immobilizing the chitosan film on polymer surfaces have been reported in the past 20 years. While authors have used various substrates ranging from polyolefins and PTFE to polysaccharide fabric, the key conclusion is that the substrates cannot bind chitosan. The polymer substrates need to be pretreated, and a widely used technique is the application of nonequilibrium gaseous plasma. Usually, oxygen, Ar, and air plasmas were used. Gaseous plasma consists of species capable of forming irreversible modifications. Positively charged ions of noble gases and vacuum ultraviolet radiation break bonds in the surface film of polymers. The dangling bonds then react with oxygen to form polar, negatively charged surface functional groups. The bond breakage could be avoided with direct exposure to oxygen species in nonequilibrium gaseous plasma, such as low-energy positively charged oxygen ions, neutral oxygen atoms, and excited neutral oxygen molecules or atoms. The interaction of reactive oxygen particles will cause the substitution of hydrogen on the polymer surface, leading to the formation of various groups. The authors mostly used oxygen-containing plasmas to form hydroxyl and especially carboxyl groups needed for the immobilization of chitosan.

Chitosan solution was generally prepared by dissolving it in slightly acidic water with a pH between 4 and 5. Usually, concentrations 1–2% (*w*/*v*) were used, in some cases, even very small concentrations below 0.1% (*w*/*v*). Two different procedures were used by the authors: (1) direct deposition of chitosan on plasma-treated substrates and (2) a two-step method using an additional intermediate grafted layer. A common method adopted by all authors was dipping the substrates into the chitosan solution. The incubation time was usually from 1 min to 2 h for direct chitosan deposition. In the case of a two-step method, the incubation time was usually much longer, i.e., 24 h. Direct functionalization causes the noncovalent attachment of chitosan by electrostatic interactions or hydrogen bonds. The amino groups of chitosan interact chemically with the negatively charged functional groups on the polymer surface, thus assuring chitosan immobilization. The chitosan layer may or may not be stable because some authors reported the removal of excessive chitosan. Still, all authors agree that at least a monolayer of chitosan remains on the polymer surface even after prolonged rinsing with water, which may be slightly acidic.

The direct (one-step) deposition of chitosan on plasma-treated samples was found useful by many authors. However, others preferred a two-step method via an intermediate layer to assure optimal immobilization. With the help of additional coupling agents, covalent binding of chitosan onto the intermediate layer was obtained. EDC and NHS coupling agents were usually used for covalent immobilization. 

Polyacrylic acid seems to be the most commonly used intermediate layer. Sometimes, PAA was combined with PEG. The methods of grafting PAA on the plasma-pretreated samples vary between the authors. Some reported exposure to acrylic acid vapors and are silent about further treatment, but others exposed the layer of deposited monomer to UV radiation to ensure appropriate cross-linkage. A possible method for depositing a highly cross-linked PAA is plasma polymerization. In such cases, plasma is sustained either in almost pure vapor or in a mixture of Ar and organic vapor. Plasma polymerization is based on the formatting of radicals from the precursor molecules. The radicals stick to the polymer surface and form chemical bonds if the surface has been previously functionalized with polar groups. The deposited polymer film will resemble polyacrylic acid, providing the precursor molecules are only slightly radicalized. Powerful plasmas will cause atomization of the precursor, so the film will be any hydrogenated carbon. The gaseous plasma is always a source of radiation, so the polymer film deposited by plasma polymerization is highly cross-linked and differs in composition and structure from pure polymer. Still, it is a useful method for depositing the intermediate film. 

Plasma pretreatment should cause the formation of a rather uniform film. However, the formation of chitosan in the form of nanoparticles was also reported [60]. In addition, even the spontaneous formation of chitosan nanofibers was reported when drying was performed by deep freezing, followed by classical vacuum drying at low temperatures [59].

All reported treatment procedures led to the successful immobilization of chitosan. The thickness of the deposited chitosan was rarely investigated, although it can influence the stability of the coating and its swelling properties. In reported cases, the thickness ranged from roughly a monolayer to films up to 1 mm. A wide range of wettability of chitosan-coated samples was mentioned, from almost superhydrophilic to hydrophobic surfaces, depending on the immobilization method and morphological substrate characteristics (foils and fibers). Too hydrophobic of a chitosan coating may influence the application of chitosan in particular applications, such as the swelling and formation of chitosan-based hydrogels. Nevertheless, the wettability of materials with a chitosan coating was more or less improved compared to nontreated substrates. Many authors also tested the properties of chitosan-coated substrates for future applications and reported excellent antibacterial properties and improved biocompatibility and hemocompatibility, as well as good barrier properties. These results clearly prove chitosan’s usefulness in medical or food packaging applications.

In summary, chitosan coatings can be successfully deposited using different plasma treatment methods with or without additional coupling agents and can be useful in many applications. The challenge yet to be tackled is the deposition of a stable coating capable of swelling upon incubation with water because the hydrophilicity of the chitosan layer is still not optimal. Another aspect for the future is the development of so-called intelligent chitosan hydrogels that can respond to factors of external environments such as temperature, pH, and electric field. For medical applications, in vivo and in vitro investigations are also needed (e.g., scaffolds for regenerative medicine and tissue engineering, application for repair of the central nervous system, and wound healing). Moreover, exact mechanisms of the chitosan antibacterial activity are still not explained.

## Figures and Tables

**Table 1:** Summary of processing parameters and surface finish reported for direct chitosan immobilization on plasma-treated substrates. Abbreviations in the table are defined in the notes below.

Reference and Year	Substrate Material	Type of Discharge	Type of Gas	Gas Pressure	Treatment Time	Chitosan Concentration and Incubation Time	WCA of Untreated Substrate	WCA of Treated Substrate	WCA of Chitosan—Coated Samples	Thickness of Chitosan
[58] 2002	Linear LDPE	Ion implantation, RF + pulsed DC	O_2_	0.17 Pa	60 s	3% (*w*/*v*); deposited and dried	86°	6°	N/A	50 µm
[59] 2015	PPC microfibers	RF CCP	O_2_	0.04 Pa	36 s	0.03–0.07 mg/mL; 2 min	122°	92°	53°	N/A
[60] 2018	PS	DC	Ar, O_2_, air	2 Pa	5–30 min	1% (*w*/*v*); 5 min	78°	~40°	80–92°	N/A
[61] 2019	PE, PP	MW-surfatron	O_2_	50 Pa	60 s	2% (*w*/*v*); N/A	108, 109°	30, 37°	42, 45°	~1 mm (estimated from its mass 0.1 g/cm^2^)
[62] 2018	PLA fibers	RF	O_2_	N/A	30–1800 s	1% (*w*/*v*); 1 min	N/A	N/A	N/A	30–100 nm
[63] 2013	Cotton gauze	DBD	He	10^5^ Pa (1 bar)	N/A	2–7%, electrospinning, 2 h	N/A	N/A	N/A	Nanofibers with a diameter of 250 nm
[64] 2014	PP	Atmospheric DBD	Air	10^5^ Pa (1 bar)	180 s	2% (*w*/*v*); 1 min	92°	68°	39°	N/A
[65] 2017	PE fibers	DBD	Ar/O_2_ (90:10)	10^5^ Pa (1 bar)	40–140 s	0.7% (*w*/*v*); N/A	114°	N/A	81–95°	N/A, very thin
[66,84] 2021	PLA	MW	Ar	10^5^ Pa (1 bar)	N/A	1% (*w*/*v*); 10 s	85°	55°	N/A	N/A
[67] 2014	PET	RF ICP	O_2_ or CO_2_	75 Pa	30 s	1.5% (*w*/*v*); 72 h	92°	28° or 35°	N/A	N/A
[68] 2013	PE	Corona	Air	10^5^ Pa (1 bar)	N/A	1 wt %, N/A	N/A	N/A	N/A	30 μm
[69] 2018	PEEK	RF CCP	Air followed by N_2_	20 Pa	1 min + 1 min	0.1 mg/mL, deposited and dried	67°	5°	52°	N/A
[70] 2020	PET	DC or AC	Air	20 Pa	10–60 s	1% (*w*/*v*); 2 h	80°	10° or 17°	~50°	N/A
[42] 2021	PET, PDMS	Torch	Chitosan	N/A	1 run	N/A	N/A	N/A	N/A	N/A
[81] 2022	Meliflex XP polyolefin	RF	H_2_ followed by O_2_	20 Pa 45 Pa	30 s 2 s	2, 2.5% (*w*/*v*); 20 min	96–103°	37–46°	82–100°	N/A

**Table 2:** Summary of processing parameters and surface finish reported for chitosan immobilization on plasma-treated substrates via an intermediate layer *.

Reference	Substrate Material	Type of Discharge	Type of Gas	Gas Pressure	Treatment Time	Intermediate Step (Grafting and Coupling Agent)	Chitosan Concentration and Incubation Time	WCA of Untreated Substrate	WCA of Treated Substrate	WCA of Intermediate Layer	WCA of Chitosan-Coated Samples	Thickness of Chitosan
[71] 2003	Nonwoven PE	MW	O_2_	33 Pa	10 s	AA and EDC	0.3% (*w*/*v*), 24 h	N/A	Small	N/A	N/A	N/A
[72] 2005	PU membrane	RF CCP	O_2_	Low	20–150 s	AA and EDC/NHS	0.25 mg/mL; 24 h	68°	N/A	N/A	50°	N/A
[73] 2006	PET	RIE, RF CCP	Ar	Low	180 s	AA and DECH	0.3% (*w*/*v*), 24 h	75°	N/A	34°	21°	N/A
[80] 2010	PVC	DCSBD	Air	10^5^ Pa (1 bar)	15 s	AA and EDAC	1% (*w*/*v*), 20 min	86°	65°	46°	63°	N/A
[79] 2012	LDPE	DCSBD	Air	10^5^ Pa (1 bar)	15 s	AA and EDAC	1% (*w*/*v*), 24 h	99°	77°	67°	69°	N/A
[68] 2013	PE	Corona	Air	10^5^ Pa (1 bar)	N/A	No grafting; EDC/NHS	1 wt %, N/A	N/A	N/A	N/A	N/A	25 μm
[74] 2015	LDPE	DC, magnetized	Ar	20 Pa	300 s	AA, PEG, no agent	1% (*w*/*v*), 1 min	95°	57°	22°	16°	N/A, very thin
[75] 2016	PP	DBD	Ar	10^5^ Pa (1 bar)	60 s	AA, PEG, and EDC	1% (*w*/*v*), 30 min	92°	65°	20°	16°	N/A, very thin
[76] 2018	PCL fibers	RF CCP	Ar	80 Pa	30 s	NHSMA, no agent	0.5–1% (*w*/*v*) Overnight	N/A	N/A	N/A	N/A	N/A, very thin
[77] 2018	PTFE	DBD	N_2_/H_2_ (95:5)	10^5^ Pa (1 bar)	45 s	GA, PEGb, or PA and EDAC	2% (*w*/*v*), 3 h	100°	N/A	N/A	50–60°	N/A
[78] 2019	LDPE	DBD	Ar +PEG and AA vapour	10^5^ Pa (1 bar)	60 s + 5 min	AA, PEG	1% (*w*/*v*), 30 min	95°	13°	13°	0°	N/A, thin
[43] 2019	PU	RF CCP	N_2_	20 Pa	120 s	AA and EDC/NHS	0.1%, 8 h	80°	20°	45°	85°	N/A
[85] 2020	PLA	PECVD	HDMSO/O_2_	N/A	30, 60 s	SiO_x_	1% (*w*/*v*)	51°	N/A	34°	N/A	40 μm
[42] 2021	PLA	RF	N_2_	40 Pa	N/A	EDC	1.3% (*w*/*v*)	89.7°	N/A	N/A	73.5°	N/A
[44] 2022	PLA	RF CCP	Air	60 Pa	60 s	AA and EDC and NHS	N/A	82°	46°	N/A	65°	N/A

Abbreviations: AA—acrylic acid, CCP—capacitively coupled plasma, DBD—dielectric barrier discharge, DC—direct current, DCSBD—diffuse coplanar surface barrier discharge, DECH—1-(3-dimethylaminopropyl)-3-ethyl-carbodiimide hydrochloride, EDAC—N-(3-dimethylaminepropyl)-N’-ethylcarbodiimide hydrochloride, EDC—1-ethyl-3-(3-dimethylaminopropyl) carbodiimide hydrochloride, HDMSO—hexamethyldisiloxane, ICP—inductively coupled plasma, LDPE—low-density polyethylene, MW—microwave, NHS—N-hydroxysuccinimide, NHSMA—N-hydroxysuccinimide ester, PCL—polycaprolactone, PE—polyethylene, PECVD—plasma-enhanced chemical vapor deposition, PEEK—polyetheretherketone, PEG—polyethylene glycol, PET—polyethylene terephthalate, PLA—polylactic acid, PP—polypropylene, PPC—polypropylene carbonate, PS—polystyrene, PTFE—polytetrafluorethylene, PU—polyurethane, PVC—polyvinylchloride, RF—radiofrequency, RIE—reactive ion etching, WCA—water contact angle.

## Data Availability

All collected and analyzed data are presented in this manuscript.
